# Solid-like heat transfer in confined liquids

**DOI:** 10.1007/s10404-017-1980-x

**Published:** 2017-08-24

**Authors:** Michael Frank, Dimitris Drikakis

**Affiliations:** University of Strathclyde, Glasgow, G1 1XJ UK

**Keywords:** Heat transfer, Thermal conductivity, Confined liquid, Phonons, Density of states, Relaxation time

## Abstract

The aim of this research is to identify possible mechanisms that govern heat transport at a solid–liquid interface using molecular dynamics. The study reveals that, unlike its bulk analogue, a liquid in a nanochannel sustains long-lived collective vibrations, phonons, which propagate over longer timescales and distances. The larger phonon mean free path in nanochannels is attributed to the greater structural order of the liquid atoms and to the larger liquid relaxation time—the time in which the liquid structure remains unchanged and solid-like. For channels of height less than $10\sigma$, long-range phonons are the dominant means of heat transfer in the directions parallel to the channel walls. The present findings are in agreement with experiments, which have observed significantly increased liquid relaxation times for the same range of channel heights. Finally, it is argued that confinement introduces additional transverse modes of vibration that also contribute to the thermal conductivity enhancement.

## Introduction

Despite the increasing use of micro- and nanoflows in many fields, the effect of confinement on the properties of liquids is only partly understood. Experimental and numerical studies have shown that interactions at the solid–liquid interface, increasingly important with diminishing system dimensions, can alter the behavior of liquids. In nanochannels, the structural order of the liquid atoms has been found to increase to various extents depending on the liquid and solid properties (Bai et al. 2010; Klein and Kumacheva 1995; Sun et al. 2002; Koga et al. 1997, 2000). This in turn has an impact on the flow dynamics; liquids often exhibit increased flow rates inside nanofluidic structures such as carbon nanotubes (Walther et al. 2013; Nicholls et al. 2012; Thomas and Corry 2015). The thermodynamic properties of liquids, such as viscosity and diffusivity, also change in nanochannels (Gao et al. 1997; Liu et al. 2005; Aoun and Russo 2016; Ghorbanian and Beskok 2016; Sofos et al. 2009).

The thermal properties of micro- and nanofluidic systems are also of academic and industrial interest, as micro- and nanochannel heat sinks are increasingly being used for thermal management. The Kapitza resistance (thermal resistance at an interface), often a bottleneck of cooling devices, can be decreased by choosing lyophilic materials that are wettable by the cooling liquid (Barrat and Chiaruttini 2003; Kim et al. 2008). This is a direct result of the increasing density of the structured liquid layers next to the channel walls (Xue et al. 2004; Alexeev et al. 2015).

It is also known that the thermal conductivity of confined liquids is in general anisotropic (Liu et al. 2005; Frank et al. 2015): As we increase the length of confinement (i.e., the diameter in the case of nanotubes or channels or the height in the case of parallel surfaces), the thermal conductivity normal to the channel surface increases. On the contrary, the thermal conductivity parallel to the solid surface decreases as the length of confinement increases. The increase in the normal thermal conductivity is attributed to the diffusivity, which also increases with the length of confinement (Liu et al. 2005; Sofos et al. 2009). However, the larger values of the parallel thermal conductivity in narrower channels are not well understood. This lack of understanding motivated this study.

Molecular dynamics (MD) simulations were performed to examine the effect of confinement on the thermal properties of liquids. We found that in a channel of $6.58\sigma$ in height, the thermal conductivity of liquid argon in the direction parallel to the channel walls is approximately three times greater than its unconfined equivalent. As the channel height increases, the thermal conductivity decreases and plateaus to the value of bulk argon at $25.6\sigma$, where $\sigma$ is the molecular diameter of argon. The thermal conductivity enhancement in smaller channels is due to phonons, which propagate, unscattered over longer distances compared to the phonons in bulk liquids. The increase in the phonon mean free path in nanochannels is due to the greater structural order of the liquid atoms and the larger liquid relaxation time—a time window in which the liquid atoms retain their structure—during which phonon scattering events are reduced. For channel heights less than $10\sigma$, we show that the thermal conductivity is dominated by long-range phonons. Experiments very similar to the setup of our simulations observed significantly increased liquid relaxation times for the same range of channel heights (Demirel and Granick 1996). Finally, by studying the vibrational density of states, we argue that liquids in nanochannels can support additional transverse vibrational modes that are not available in bulk liquids. Using Frenkel’s theories for liquid thermodynamics (Frenkel 1946; Bolmatov et al. 2012), we attribute the availability of the transverse oscillations to the larger liquid relaxation time.

## Molecular model

MD simulations were performed using the LAMMPS Molecular Dynamics Simulator (Plimpton et al. 2007). The model consists of liquid argon confined by two parallel ideal solid surfaces. The walls of the channel are fixed perpendicular to the *y*-direction, and periodic boundary conditions are used parallel to the channel (*x*- and *z*-direction) to emulate its perpetual continuation. The dimensions in the $x$- and $z$-directions are $L_{x} = 14.14\sigma$ and $L_{z} = 16.33\sigma$, respectively. Far from being arbitrary, these values ensure that the thermal conductivity does not change by further increasing the size of the simulation box in the $x$- and $z$-directions. Physically, this convergence of the thermal conductivity implies that the system is large enough to accommodate for the largest phonon mean free path of the liquid in the corresponding directions. $L_{y}$ is the distance separating the walls in the *y*-direction and varies across different cases, ranging between $3\sigma$ and $25.6\sigma$. The solid particles are placed on two (111) FCC lattice planes, forming closed-group layers that minimize the gaps for liquid atoms to escape. The argon particles are initially placed randomly in the channel. The number of inserted particles is adjusted depending on the volume of the system to realize a constant density of $0.84\rho^{*}$, where $\rho^{*}$ is the reduced density.

The liquid particles interact through Lennard-Jones (LJ) potentials where the molecular diameter and the depth of the potential well used are $\sigma_{ll} = 1.0\sigma$ and $\varepsilon_{ll} = 1.0\varepsilon$, respectively. The wall atoms are fixed onto their initial lattice sites using spring potentials of stiffness $\kappa = 500\varepsilon \sigma^{2}$. The solid–liquid interactions are also modeled through Lennard-Jones potential with $\varepsilon_{wl} = 0.8\varepsilon$ and $\sigma_{wl} = 0.75\sigma$.

The system temperature is regulated solely through the walls by rescaling the atomic velocities of the solid atoms (Kim et al. 2008). We have not tampered with the temperature of the liquid in any way as that has been shown to introduce unrealistic behavior (Bernardi et al. 2010; Basconi and Shirts 2013; Thomas and Corry 2015); instead the liquid atoms conduct heat to and from the walls through natural interactions/collisions.

The system is brought into thermal equilibrium at a temperature of $0.71T^{*}$, where $T^{*}$ is the reduced temperature; this temperature, along with the aforementioned density, corresponds to the liquid state of argon (Allen and Tildesley 1989; Sarkar and Selvam 2007). The system evolves within the micro-canonical (NVE) ensemble with a timestep of $\tau \approx 2fs$. After an initial equilibration phase of $10^{6} \tau$, the system is sampled for a further $4 \times 10^{7} \tau$ for the calculation of the thermal conductivity. The thermal conductivity is calculated using the Green–Kubo relation, which correlates the thermal conductivity to the integral of the heat flux autocorrelation function (HFACF) of the system in equilibrium.

Previous studies have shown that the thermal conductivity in nanochannels is anisotropic between the directions parallel and normal to the channel walls (Liu et al. 2005; Frank et al. 2015). The behavior of the normal thermal conductivity is well understood: As the channel width increases, so does the molecular diffusion, leading to an increased thermal conductivity normal to the channel walls. Here, we are concerned with the thermal conductivity in the parallel directions, whose physical origins are, to the best of our knowledge, absent from the literature. Therefore, any reference to the thermal conductivity of the confined liquid is strictly referring to its component in the *x*-direction (the *z*-direction yielded identical results).

## Results and discussion

**Fig. 1:**
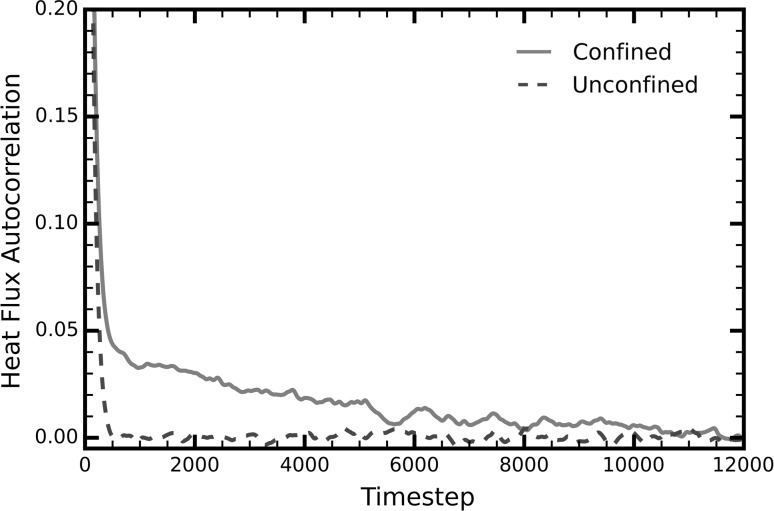
Heat flux autocorrelation function in the *x*-direction for unconfined liquid argon (*blue*) and for liquid argon in a nanochannel (*red*). In general, the function shows how an average energy perturbation in the system dissipates over time. For bulk argon (*blue*), energy disturbances dissipate monotonically within $400$ timesteps. For argon in a nanochannel (*red*), energy perturbations dissipate in two stages and vanish after $10000$ timesteps. This shows that phonons in the system can propagate unscattered for longer (colour figure online)

Our results reveal a different dissipation pattern of energy perturbations between confined and unconfined liquids (Fig. 1). In bulk argon, energy disturbances dissipate monotonically within a short time frame—approximately $400\tau$—reflecting the diffusive nature of heat transfer in liquids. Under confinement, however, energy perturbations in the system follow a two-stage decay: a short-lived component coinciding with its unconfined counterpart and a more gradual decrease extending in time beyond $10000\tau$.

Previous studies (McGaughey and Kaviany 2004; Kaburaki et al. 2007) have shown that the transition from liquid or amorphous solid to crystal argon is accompanied by the exact same change in the HFACF as that observed here between unconfined and confined liquid argon. Other crystalline materials have shown similar trends (Chen et al. 2010; Yang et al. 2014). The authors of these studies attribute the phenomenon to the existence of long-range phonons in perfect crystals, which carry energy over longer distances and timescales, causing the HFACF to decay more slowly.

**Fig. 2:**
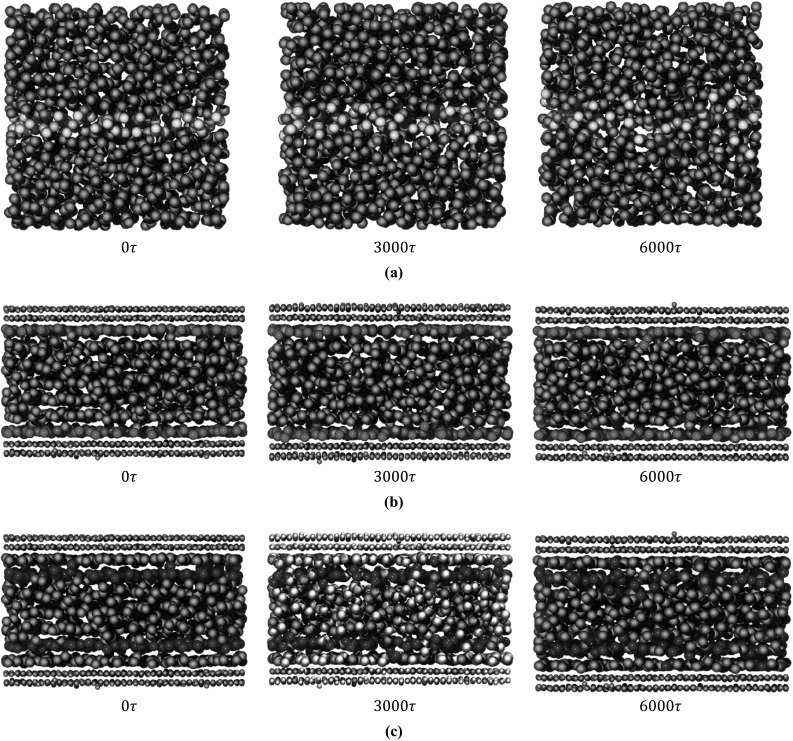
Evolution of liquid particles in time. **a** A random selection of atoms in bulk liquid. The atoms diffused significantly even after $3000\tau$. After $6000\tau$, the initial configuration is no longer recognizable. **b** Evolution of the first liquid layer next to each wall (*colored in red*). The atoms retain their structure even after $6000\tau$ with very little diffusion. **c** Evolution of the second layer next to each wall (*colored in blue*). After $3000\tau$, the atoms retain their structure. However, more diffusion is observed compared to the first layer. After $6000\tau$, although the layers are still recognizable, the liquid atoms have diffused significantly (colour figure online)

**Fig. 3:**
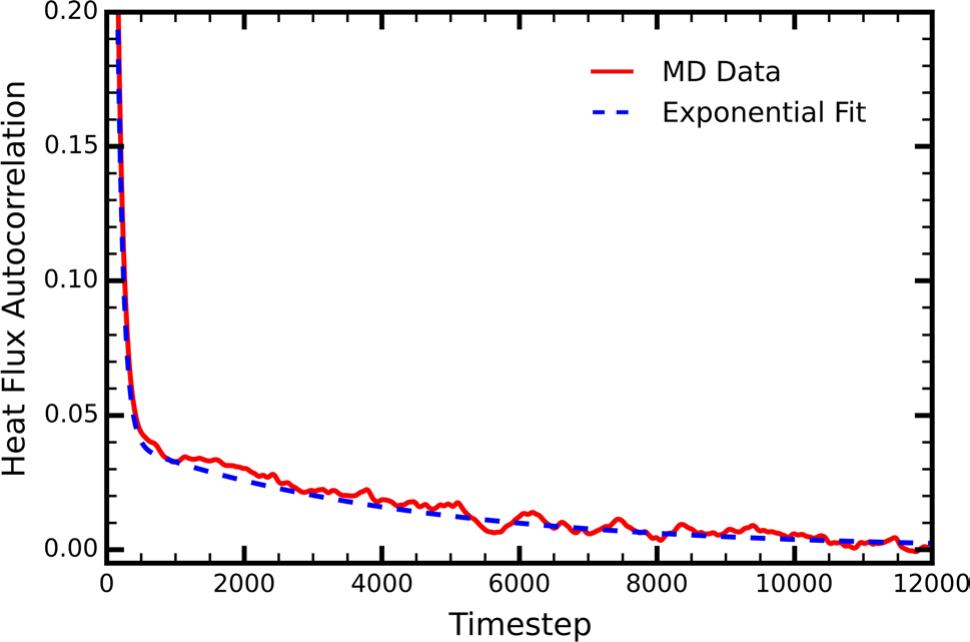
Heat flux autocorrelation function (HFACF) of a liquid in a 6.58*σ* channel as obtained directly by the MD data (*red*, *solid line*) and as obtained by fitting a sum of two exponential functions onto it (*dashed blue line*) (colour figure online)

In a similar manner, we attribute the two-stage decay of liquids in nanochannels to the existence of long-range phonons. In bulk liquids, phonons scatter frequently due to anharmonicities originating from the structural disorder and constant deformation of liquid atoms (Fig. 2a). However, experiments have shown that in nanochannels, the relaxation time of liquids increases by up to $10^{7}$ times the Brownian motion in bulk liquids (Hu et al. 1991). During this relaxation time, the liquid atoms retain their structure (qualitatively shown in Fig. 2b, c) and phonon scattering events are reduced. Furthermore, the arrangement of the liquid atoms in nanochannels into neat, solid-like planes parallel to the channel walls further reduces anharmonicities. An accurate analogy is the comparison between amorphous and crystalline solids, i.e., crystals usually have greater thermal conductivities due to the highly symmetric configuration of their atoms. As Fig. 2c (and to a lesser extent Fig. 2b) shows, there is still a significant amount of diffusion in nanochannels, which justifies the short-lived component of the decay. To quantify the effect of long-range phonons on the thermal properties of the liquid, we decomposed the thermal conductivity into its short- and long-range components by fitting a sum of two exponential functions onto the HFACF (Fig. 3) (McGaughey and Kaviany 2004; Che et al. 2000).

**Fig. 4:**
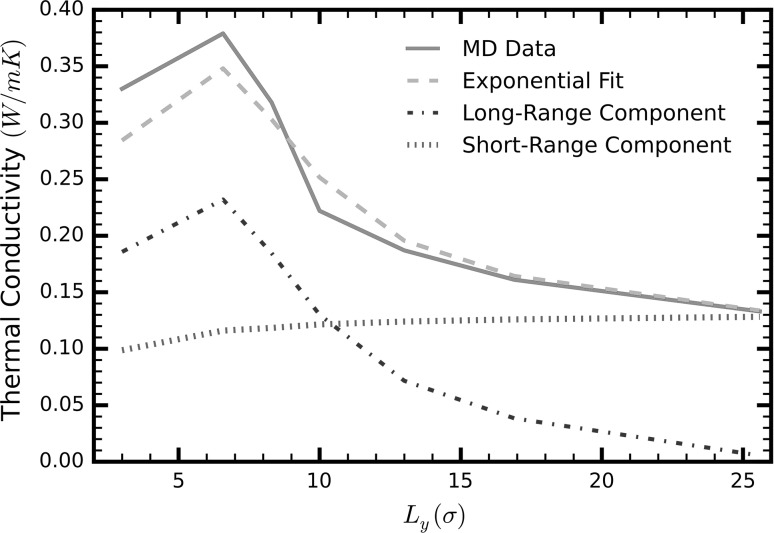
Thermal conductivity of liquid argon in a nanochannel in the *x*-direction as a function of the channel width. The four curves represent the property as obtained by integrating the heat flux autocorrelation function using MD data (*solid green line*). Specifically, this was achieved by integrating a sum of two exponential functions fitted on the HFACF (*dashed red line*), and the individual short (*dotted purple line*)- and long (*blue, dash dot line*)-range effects obtained by the individual exponential function (colour figure online)

**Fig. 5:**
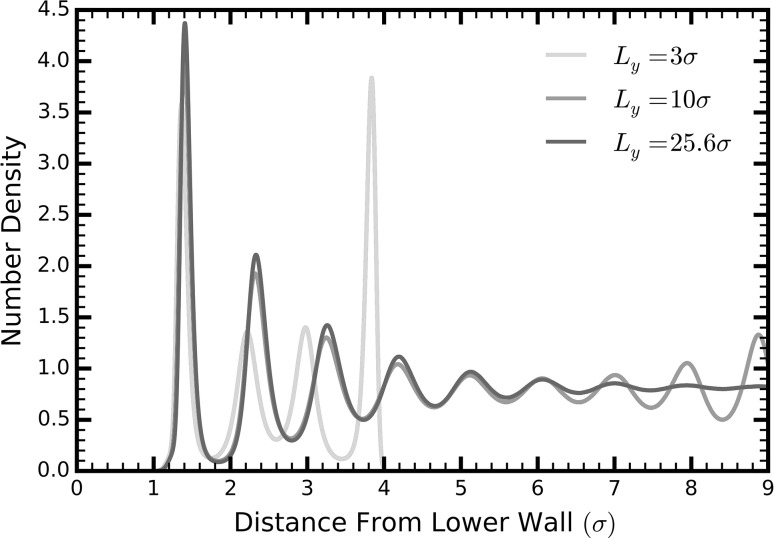
Number density of the liquid for three different channel heights. Up to a channel height of approximately $10\sigma$, the liquid is stratified across the entire height of the channel (*blue line*). For a channel of height $25\sigma$, the liquid density becomes uniform and bulk-like at the center of the channel. When the channel is only $3\sigma$ in height, only four layers form in the channel with a different oscillatory pattern than for larger channels. This is due to the liquid atoms being affected by the presence of walls (colour figure online)

Figure 4 shows that, with the exception of the first point, the total thermal conductivity decreases with increasing channel height converging to $0.132\,\,{\text{W/mK}}$, i.e., the experimentally measured value for bulk liquid argon (Muller-Plathe 1997). We observe a maximum value of $0.38\,\,{\text{W/mK}}$ at a confinement of $6.58\sigma$, an almost threefold enhancement compared to the liquid’s bulk form at the same temperature. We attribute the slightly decreased thermal conductivity of the first point—approximately $3\sigma$ channel height—to the overly restricted motion of the liquid particles. This restriction is shown by the different oscillatory pattern of the density profiles of the system compared to those of larger channel heights (red curve in Fig. 5).

For channel heights less than $10\sigma$, the thermal conductivity is dominated by long-range phonons (dashed dot, blue line in Fig. 4). Experiments using a setup very similar to that of our simulations—Lennard-Jones-like liquids confined in atomically smooth walls—observed a significant increase in the liquid relaxation time for the same range of channel heights (Demirel and Granick 1996). This supports our argument that the long-range phonons are enabled by a larger liquid relaxation time.

As the channel height increases to approximately $25\sigma$, the long-range effects vanish. Conversely, the short-range component (dotted, purple line in Fig. 4) of the thermal conductivity increases with the height of the channel, reaching a plateau at approximately $25\sigma$.

We attribute the increase in the liquid relaxation time to the structural order of the liquid in nanochannels (Fig. 5). For channel heights below $10\sigma$, the liquid is solid-like and stratified across the entire channel height. We believe that the increased order in smaller channels further reduces the anharmonicities in the system. At larger scales, however, the density at the center of the channel plateaus to that of bulk liquid argon, an indication that the liquid atoms behave diffusively and are under constant deformation.

**Fig. 6:**
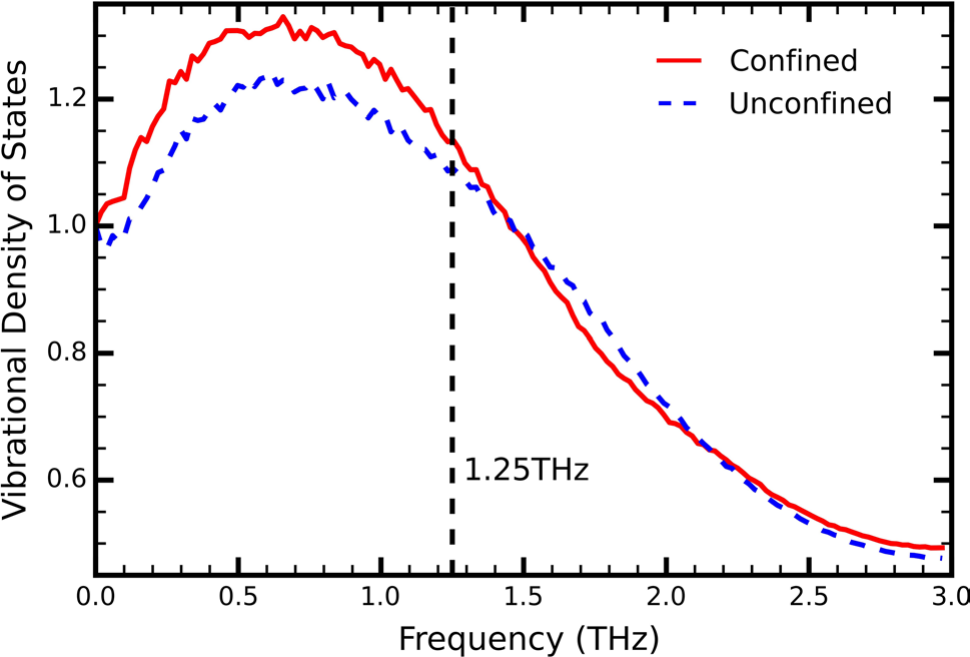
Vibrational density of states (VDOS) for confined (*red, solid line*) and unconfined (*blue, dashed line*) liquids. The *black vertical line* marks the inverse of the phonon relaxation time of bulk argon, as measured by the heat flux autocorrelation function (colour figure online)

To further compare the dynamics between the bulk and confined liquid, we consider the normalized vibrational density of states (VDOS), obtained by Fourier transforming the velocity autocorrelation function of the liquid particles (Fig. 6). The non-zero value at zero frequency is the diffusive vibrational mode and is a characteristic of the VDOS of liquids and gases. Although confinement doesn’t seem to excite different vibrational frequencies, it introduces additional vibrational states for the same range of frequencies, which further enhance the thermal conductivity.

According to Frenkel (1946), liquids can support transverse oscillations whose frequencies are greater than the inverse of the liquid relaxation time $${\raise0.7ex\hbox{$1$} \!\mathord{\left/ {\vphantom {1 {\tau_{F} }}}\right.\kern-0pt} \!\lower0.7ex\hbox{${\tau_{F} }$}}$$. If we approximate the liquid relaxation times of our systems by the phonon relaxation time obtained from the HFACF then for confined liquids, we get $${\raise0.7ex\hbox{$1$} \!\mathord{\left/ {\vphantom {1 {\tau_{F} }}}\right.\kern-0pt} \!\lower0.7ex\hbox{${\tau_{F} }$}} = 0.05\,\,{\text{THz}}$$, whereas for the bulk liquid, we get $${\raise0.7ex\hbox{$1$} \!\mathord{\left/ {\vphantom {1 {\tau_{F} }}}\right.\kern-0pt} \!\lower0.7ex\hbox{${\tau_{F} }$}} = 1.25\,\,{\text{THz}}$$. Indeed, the major discrepancies between the VDOS of the two systems lie within this interval; beyond the frequency $${\raise0.7ex\hbox{$1$} \!\mathord{\left/ {\vphantom {1 {\tau_{F} }}}\right.\kern-0pt} \!\lower0.7ex\hbox{${\tau_{F} }$}} = 1.25\,\,{\text{THz}}$$, the two curves are coincident with each other, within statistical accuracy. Therefore, we attribute the increased VDOS in confined liquids to transverse modes of vibration enabled by the increased relaxation time—or more precisely, the decrease in the inverse of the relaxation time. Although in this study the difference in the VDOS between the confined and unconfined liquids is relatively small, we believe that in liquids with different molecular mass, intermolecular interactions and molecular shape, the effect could be greater.

Finally, in atomically perfect nanotubes, as opposed to parallel surfaces, the thermal conductivity of water follows similar trends (Liu et al. 2005), suggesting that a smoothly varying channel geometry does not significantly affect phonon propagation. However, a number of studies have shown that irregular surface roughness, inherently found on many surfaces, breaks the structured layers close to the channel walls (Papanikolaou et al. 2016, 2017). This, in turn, has implications on liquid properties such as viscosity (Papanikolaou et al. 2017). Therefore, as a concluding remark and prospect for future work, we speculate that surface roughness will increase the frequency of scattering events, reducing the value of the thermal conductivity.

## Conclusions

We have performed MD simulations to study how the thermodynamic behavior of liquid argon changes under confinement. We have shown that the thermal conductivity in the direction parallel to the solid surfaces is enhanced almost three times in a nanochannel of height $6.58\sigma$ compared to the equivalent unrestricted liquid. As the channel height increases, the thermal conductivity decreases and converges to the value of bulk argon. We attribute this enhancement to the existence of long-range phonons available in the system due to the increased structural order and larger relaxation time of liquids under confinement. The results show that long-range phonons are the dominant means of heat transfer in nanochannels of height less than $10\sigma$. Experiments observed significantly increased liquid relaxation times for the same range of channel heights, further supporting our argument. The effect of long-range phonons on the thermal conductivity vanishes completely for channels with height greater than $25\sigma$. Finally, by comparing the vibrational density of states between confined and unconfined liquids, we concluded that confinement introduces additional transverse oscillations. We attribute the extra transverse vibrational modes to the increase in the relaxation time such that its inverse is smaller than the vibrational frequencies of the system.
